# Triple negative breast cancer: special histological types and emerging therapeutic methods

**DOI:** 10.20892/j.issn.2095-3941.2019.0465

**Published:** 2020-05-15

**Authors:** Lu Cao, Yun Niu

**Affiliations:** ^1^Department of Pathology, Tianjin Medical University Cancer Institute and Hospital, National Clinical Research Center for Cancer, Key Laboratory of Cancer Prevention and Therapy, Tianjin, Tianjin’s Clinical Research Center for Cancer, Tianjin 300060, China; ^2^Department of Breast Cancer Pathology and Research Laboratory, Tianjin Medical University Cancer Institute and Hospital, National Clinical Research Center for Cancer, Key Laboratory of Cancer Prevention and Therapy, Tianjin, Tianjin’s Clinical Research Center for Cancer, Tianjin 300060, China

**Keywords:** Triple negative breast cancer, pathological subtype, androgen receptor, immunotherapy, targeted therapy

## Abstract

Triple negative breast cancer (TNBC) is a complex and malignant breast cancer subtype that lacks expression of the estrogen receptor (ER), progesterone receptor (PR) and human epidermal growth factor receptor 2 (HER2), thereby making therapeutic targeting difficult. TNBC is generally considered to have high malignancy and poor prognosis. However, patients diagnosed with certain rare histomorphologic subtypes of TNBC have better prognosis than those diagnosed with typical triple negative breast cancer. In addition, with the discovery and development of novel treatment targets such as the androgen receptor (AR), PI3K/AKT/mTOR and AMPK signaling pathways, as well as emerging immunotherapies, the therapeutic options for TNBC are increasing. In this paper, we review the literature on various histological types of TNBC and focus on newly developed therapeutic strategies that target and potentially affect molecular pathways or emerging oncogenes, thus providing a basis for future tailored therapies focused on the mutational aspects of TNBC.

## Introduction

Breast cancer is one of the most common malignancies and one of the main causes of cancer-related death among women^1,2^. Triple negative breast cancer (TNBC) accounts for approximately 10%–15% of all breast cancers and is characterized by a lack of expression of the estrogen receptor (ER), progesterone receptor (PR), and human epidermal growth factor receptor 2 (HER2)^3^. Compared with hormone receptor-positive breast cancers, TNBC has fewer treatment options, and its prevalence in younger and obese women is high, as is the recurrence and death rate among patients 3–5 years after surgery^4^. Furthermore, TNBC is closely associated with BRCA1/2 mutation status, and more than 20% of patients with TNBC are carriers of such mutations^5^.

Biologically, TNBC tumors tend to be more aggressive and larger, to have a higher oncological grade, and to be accompanied by lymph node metastasis^6^. Because of the absence of well-defined molecular targets, TNBC treatment relies on chemotherapy, mainly an anthracycline- and taxane-based regimen^7^. Despite the higher rates of clinical response to neoadjuvant chemotherapy, patients with TNBC still have higher rates of distant recurrence and poorer prognosis than women with other breast cancer subtypes. Once metastasized, TNBC is likely to involve critical visceral organs and to result in a significantly shorter median overall survival than that in other subtypes^8^. At present, TNBC therapeutic schemes heavily depend on taxanes and anthracycline because of the high heterogeneity of TNBC. The discovery and study of effective therapeutic targets remains a major priority for medical researchers^9^.

In this review, we discuss several histological classifications and TNBC subtypes, most of which have relatively good prognosis. We also discuss the current status of targeted therapy in clinical trials and the currently promising new targets in advanced TNBC that have been identified through preclinical and/or clinical research.

## Rare histological types of TNBC

TNBC is a molecular classification; if a breast tumor is negative for expression of ER, PR, and HER2, it is defined as TNBC. The histological typing of breast cancer is pathological and is based on various observations of the histological morphology of breast cancer. In most cases, histologically classified breast cancers include various molecular types of breast cancer. For example, the most common histopathological type of invasive breast cancer may be TNBC but could also be any type of molecular breast cancer. However, some rare histomorphologic subtypes of breast cancer frequently have a ER-, PR-, and HER2-negative molecular phenotype. Patients diagnosed with these rare histomorphologic subtypes often have better prognosis than those with typical TNBC. In this section, we describe several rare TNBC subtypes, most of which have relatively good prognosis.

### Adenoid cystic carcinoma (ACC)

**Figure 1:**
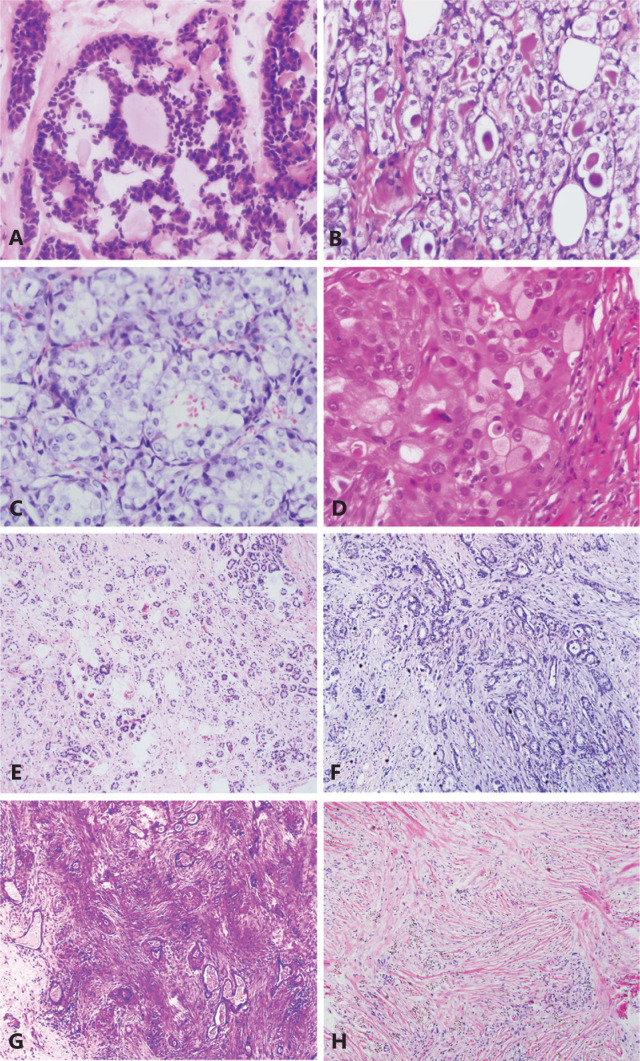
Representative H&E images of rare triple negative breast cancer. (A) Adenoid cystic carcinoma (400×). Most adenoid cystic carcinoma cells surround spherules of basement membrane material constituting pseudolumina. (B) Secretory carcinoma (400×). Abundant intracellular and extracellular secretory material is a typical feature of secretory carcinoma. (C) Acinic cell carcinoma (400×). An example of acinic cell carcinoma with abundant clear cytoplasm. (D) Carcinoma with apocrine differentiation (400×). The example shows cells with abundant, granular, intensely eosinophilic cytoplasm and enlarged nuclei with prominent nucleoli (type A cells). (E) Microglandular adenosis (100×). Haphazard proliferation of small round glands with open lumina composed of a single layer of flat to cuboidal epithelial cells is seen. (F) Carcinoma arising in microglandular adenosis (100×). Carcinoma arising in microglandular adenosis shows a more complex and disordered architecture. (G) Low-grade adenosquamous carcinoma of the breast (200×). The example shows well-developed glandular and tubular formation closely admixed with solid nests of squamous cells on a spindle-cell background. (H) Low-grade fibromatosis-like metaplastic carcinoma (200×). An example of low-grade fibromatosis-like metaplastic carcinoma composed of deceptively bland-looking spindle cells arranged in wavy, interlacing fascicles is shown.

ACC of the breast is a unique subtype of breast cancers that generally exhibit basal-like, triple-negative breast features. ACCs are generally low-grade and show indolent clinical behavior^10^. ACC is a salivary gland-type of breast cancer and is a rare subtype of TNBC. Its incidence accounts for less than 0.1% of all primary breast carcinomas^11^. ACCs are usually circumscribed tumors with a mean size of 3.0 cm and a range from 0.5 to 12 cm^12^. Approximately half of ACCs are found in the subareolar area. Pain or tenderness is non-specific and is found in rare cases^13^. Breast ACC histological features include both epithelial and myoepithelial elements and are similar to those of salivary gland ACC^14^. Neoplastic cells are polarized around 2 types of structures: true glandular spaces and pseudolumina. Ture glandular spaces are surrounded by luminal cells, and are small and difficult to see. These pseudolumina vary in shape, but are mostly round, and contain a myxoid acidic stromal substance (**Figure 1A**). However, patients with ACC of the breast have better prognosis than those with ACC of the salivary gland. On the basis of broad molecular and genetic analysis studies, ACCs typically do not express ER, PR, and HER2, but do express basal or myoepithelial markers such as cytokeratins (CKs) 5, 5/6, 14, and 17^15^. Approximately 65% of ACC cases overexpress EGFR. ACC’s molecular and genetic features indicate the presence of a MYB-NFIB fusion gene, as a result of the specific chromosomal translocation t (6; 9)^16^.

ACC is a low-grade malignant tumor associated with an overall excellent prognosis better than those of other TNBCs. The 5-year and 10-year survival rates are > 95% and 90%, respectively. ACC rarely spreads *via* the lymphatic system or distant metastasis. On the basis of its indolent clinical behavior and relatively good prognosis, ACC of the breast is usually cured by surgery, such as extensive resection or quadrantectomy with or without radiotherapy^17^.

### Secretory carcinoma

Secretory carcinoma is a very rare pathological type of invasive breast cancer, which accounts for < 1% of all breast cancers^18,19^. In general, secretory carcinoma has a triple-negative molecular phenotype and low-level clinical process, and is associated with favorable prognosis^20^. Secretory carcinoma is often called “juvenile carcinoma” because it is common in children and adolescents. The median age of presentation is 25 years (with a range of 3–87 years). Secretory carcinoma developing to systemic metastases is extremely rare^21^.

Most secretory carcinomas contain 3 patterns: microcystic, solid, and tubular (**Figure 1B**). The microcystic pattern is composed of small cysts mimicking thyroid follicles. The tubular pattern shows luminal containing secretions. Although secretory carcinomas are well-circumscribed in microscopy, its pushing of borders, areas of clear invasion, and cribriform-like adenoid structures can be easily confused with *in situ* carcinomas in needle biopsies. Positive histochemical staining of the intracellular and extracellular secretory material with periodic acid-Schiff or Alcian blue produces consistent findings. Positive immunohistochemical (IHC) staining of the epithelial membrane antigen and S-100 in tumor cells can also be helpful in diagnosis. Del Castillo et al.^22^ have demonstrated that secretory carcinoma is associated with a characteristic balanced translocation, t (12; 15), which creates an ETV6-NTRK3 gene fusion. Secretory carcinoma is primarily treated by surgery. Sentinel lymph node biopsy is recommended because of the reported axillary metastases (incidence of 30% in patients with tumors larger than 2 cm)^23^.

### Acinic cell carcinoma (ACCA)

ACCA of the breast is a rare tumor recognized as a subtype of TNBC^24^. Roncaroli et al.^25^ first reported this carcinoma in 1996, and the true incidence of ACCA is unknown because of the lack of a large series of studies. ACCA cells feature clear “hypernephroid” cytoplasm and may predominate (**Figure 1C**). Moreover, the IHC profile of breast ACCA has many features in common with salivary gland ACCA. Both frequently express S-100, lysozyme, amylase, A1-ACT, and show periodic acid-Schiff staining, and, in contrast to typical ACCA secretory carcinoma, neither frequently shows the t (12; 15) ETV6-NTRK3 rearrangement. Recently, some studies have posited that ACCA may be associated with mutations in the TP53 and MLL3 genes, and the amplification of the FOXA1 gene. Axillary lymph-node metastases may be observed in ACCA, but few of patients have been reported to have died as a consequence of this tumor^26^.

### Carcinoma with apocrine differentiation

Carcinomas with apocrine differentiation may comprise any invasive cancer with the cytological features of apocrine glands. The coding of its tumors depends upon the type of primary infiltration. Local apocrine gland differentiation is a common feature of non-specific invasive carcinoma (NST) and is present in some particular types^27^. In addition, approximately 4% of invasive breast carcinomas show extensive apocrine differentiation. Carcinomas with apocrine differentiation have cells with enlarged nuclei with prominent nucleoli, and abundant granular, eosinophilic cytoplasm with diastase-resistant periodic-acid-Schiff positivity (type B cells), abundant foamy cytoplasm (type B cells), or a combination of both (**Figure 1D**). Type A and type B cells usually have GCDFP15 and AR positive expression, and ER and PR negative expression. Comparative genomic hybridization has indicated differentiation in breast cancer with apocrine differentiation, with chromosomal gains of 1p, 1q, and 2q, and losses of 1p, 12q, 16q, 17q, and 22q. Microarray studies of gene-expression have indicated that a carcinoma with apocrine differentiation is characterized by increased androgen signaling pathways and often overlaps with the “HER2 group”^28^. Importantly, the molecular apocrine subtype defined by gene-expression microarray analysis is not equivalent to apocrine differentiation in breast cancer. Approximately half the carcinomas with apocrine differentiation show this molecular feature, but overall, carcinomas with apocrine differentiation do not form a unique cluster; the differentiation consists of “apocrine” and “luminal” molecular subtypes^29^. Studies have shown that carcinomas with apocrine differentiation have a relatively poor prognosis compared with that of NST invasive carcinomas. The androgen signaling associated with these tumors may lead to the development of new treatments in the future^30^.

### Carcinoma arising in microglandular adenosis (CAMGA)

Microglandular adenosis (MGA) is an uncommon benign disease that causes proliferative glandular lesions in the breast. These benign lesions may give rise to atypical microglandular adenosis (AMGA) and CAMGA^31^. MGA is non-lobulocentric and is composed of a layer of flat cubic epithelial cells, and myoepithelium is absent (**Figure 1E**). Although the glandular growth pattern of AMGA is retained, the epithelial cells show nuclear and architectural atypia and mitosis. The cells of MGA are typically negative for ER, PR, and HER2, and exhibit strong expression of S-100 and cytokeratin. Whether MGA is a true benign proliferation or an indolent precursor lesion remains unclear^32^. Although AMGA and CAMGA may have a background of MGA, researchers still cannot determine which examples are likely to progress in this fashion. If small glands with an infiltrating growth pattern are seen, and the architecture is more complicated and disordered under an MGA background, the possibility of CAMGA should be considered (**Figure 1F**). CAMGA treatment must be individualized on the basis of the patient’s progress of disease. The prognosis of patients with MGA is uncertain, and whether excision must be completed is controversial^33^. If MGA is accompanied by AMGA or CAMGA, it must be completely removed with a negative surgery cut edge. Some studies recommend excisional biopsy when MGA is found in a needle-core biopsy. In addition, some researchers have indicated that a primarily benign MGA lesion that is not completely removed also has a risk of MGA recurrence or even CAMGA development. Although some researchers have reported relatively good prognosis, the exact prognosis of CAMGA remains uncertain. Studies with longer follow-up periods focused on CAMGA remain needed^34^.

### Low-grade metaplastic carcinoma

Notably, beyond the 5 specific TNBCs highlighted above, additional rare types of TNBC exist, such as low-grade adenosquamous carcinoma of the breast and fibromatosis-like metaplastic carcinoma. Both are low-grade metaplastic carcinomas, which are part of a group of neoplasms characterized by differentiation of the neoplastic epithelium into squamous cells and/or mesenchymal-like the elements^35^. Low-grade adenosquamous carcinomas show well-developed glandular and tubular formation closely admixed with solid nests of squamous cells on a spindle-cell background (**Figure 1G**). Low-grade fibromatosis-like metaplastic tumors of the breast are characterized by bland spindle cells with mild or absent nuclear atypia (**Figure 1H**)^36^. Immunohistochemical analysis of metaplastic carcinomas has revealed that > 90% of these cancers are negative for ER, PR, and HER2^37^. In addition, microarray-based gene-expression profiling has demonstrated that metaplastic breast tumors are preferentially classified into the basal-like subtype^38^. Importantly, evidence suggests that, as a group, metaplastic breast cancers have lower response rates to conventional adjuvant chemotherapy and a poorer clinical outcome than other forms of TNBCs. Notably, however, independent studies suggest that low-grade fibromatosis-like carcinomas and low-grade adenosquamous carcinomas may have better clinical outcomes than other types of metaplastic breast cancer^39^.

These rarer TNBCs have a more favorable prognosis than classical TNBC types, and aggressive treatment should be avoided. A summary of the above specific pathological TNBC types is shown in **Table 1**. Importantly, carcinomas with apocrine differentiation usually have poorer prognosis than NST invasive carcinomas, possibly because of the high expression of AR in this type of breast cancer. Next, we will discuss the latest findings on AR as a therapeutic target in TNBC.

## New targets for TNBC

### Androgen receptor

Luminal androgen receptor (LAR) is a molecular subtype of TNBC that typically exhibits high expression of AR, on the basis of gene expression and cluster analysis research^40^. LAR has the lowest pathologic complete response (pCR) rate after neoadjuvant chemotherapy. Barton et al.^41^ have demonstrated that AR suppression enhances the sensitivity of chemotherapy by inhibiting the stemness of chemotherapy resistance cells. The sensitivity of LAR tumors to AR inhibition has been extensively confirmed in pre-clinical *in vitro* and xenograft research. Many retrospective studies have found that positive expression of AR is not limited to the LAR phenotype but is also expressed in other TNBC subtypes^42,43^.

Bicalutamide, a non-steroidal AR inhibitor, can competitively inhibit the combination of androgens with AR^44^. It is commonly used as a monotherapy or in combination with a gonadotropin-releasing hormone agonist for the treatment of locally advanced or metastatic prostate cancer. Gucalp et al.^45^ have performed a phase II trial study involving a group of 424 females with negative expression of ER and/or PR breast cancer to assess the efficacy of antiandrogen therapy (oral, 150 mg/d bicalutamide). AR positive expression was defined as IHC > 10% breast cancer tumor cell nuclear staining^46^. This trial resulted in a 6-month clinical benefit rate (CBR) of 19%. The median progression-free survival (PFS) was 12 weeks, and bicalutamide was well-tolerated, with no grade 4/5 treatment-related adverse events observed. Enzalutamide, a second-generation nonsteroidal antiandrogen, increases the affinity of AR five-fold over that of bicalutamide and prevents nuclear translocation of ligand-bound AR^47^. Preclinical studies have shown that enzalutamide promotes tumor cell apoptosis in LAR and non-LAR TNBC subtype cell lines, thus suggesting that enzalutamide may be available even in TNBC cell lines with weakly positive AR expression^48^. Another single-agent phase II clinical trial has assessed the safety and effectiveness of enzalutamide in patients with TNBC with advanced AR-positive expression. Seventy-five patients with TNBC were included in the trial, and the 16-week and 24-week CBR values were 35% and 29%, respectively^49^. AR inhibition should be considered as an important medicinal target for LAR or even non-LAR with AR-weak, positive TNBC, and further clinical studies on the anti-TNBC effects of bicalutamide and enzalutamide are necessary.

Molecular apocrine breast cancer (MABC), in addition to having HER2 expression, has a high registration with the LAR subtype in TNBC^50^. The transcription factor FOXA1 promotes AR signaling, thereby targeting genes; moreover, both molecules play an oncogenic role in MABC cells. AR binding regions overlap more than 98% with FOXA1 binding sites, and decreased FOXA1 expression can suppress AR signalling^51^. Extensive clinical data indicate that AR participates in many signaling pathways, such as phosphoinositide 3-kinase (PI3K)/protein kinase B (AKT)/mechanistic target of rapamycin (mTOR), MAPK, and cell cycle pathways, and interacts with many central molecules, such as FOXA1 and PTEN^52^.

LAR subtype cells frequently carry PIK3CA mutations, which make them highly sensitive to PI3K/mTOR inhibition. Lehmann et al.^53^ have studied PIK3CA gene mutations, which are more abundant in AR-positive patients with TNBC; they found mutations in 40% of AR-positive patients but only 4% of AR-negative patients with TNBC. Ample data have indicated that AR promotes PTEN gene expression in breast cancer and that targeting AR alone suppresses PTEN activity, thus activating the PI3K pathway. As a consequence, a synergistic effect occurs with the treatment with PI3K inhibitors and AR inhibitors. Lehmann et al.^53^ have demonstrated that, in combination with PI3K inhibitor GDC0941 or dual PI3K/mTOR inhibitors GDC-0980 and bicalutamide, AR inhibitors have an additional antiproliferation effect in LAR subtypes. Recently, several pre-clinical studies have demonstrated that inhibition of cell growth is possible with simultaneous inhibition of PI3K/mTOR signaling and AR in LAR tumours^54^. Combining AR-targeted therapies with other therapies may lead to breakthroughs in LAR subtype research. However, the use of AR as a therapeutic tool in breast cancer treatment remains relatively limited and is mainly applied in prostate cancer. More specific research and exploration of the heterogeneity of AR signaling in the treatment of different types of breast cancers is needed.

### Immunotherapy

Immunotherapy, an emerging but rapidly developing cancer treatment strategy, also plays an important role in TNBC tumor elimination. The immune system, through recognition of tumor-associated antigens, evokes an immune response to tumor rejection under certain conditions. Tumor-infiltrating lymphocytes (TILs) are an important cellular component in breast cancer immunotherapy, which are distributed between tumor tissue cells and adjacent stromal tissues^55^. TILs contain multiple types of immune cells, including B-lymphocytes, T-lymphocytes, and macrophages. TILs stimulate the tumor cell microenvironment to produce an immunosuppressive tumor microenvironment or an immune cancer-promoting tumor microenvironment by initiating inflammatory reactions^56^. Because current TNBC treatments mainly depend on surgery, irradiation, and cytotoxic chemotherapy to delay tumor progression and growth, the presence of TILs and immunotherapy may enable a new treatment for TNBC. Recent data demonstrate that, compared with the other histological subtypes, TNBC is a highly heterogeneous breast cancer subtype with higher expression levels of TILs. These elevated levels are predictive of the existence of an active immune system in TNBC, thus enabling increased pathological complete responses to immunotherapy^57^.

Programmed cell death protein 1 (PD-1) and its ligand PD-L1 are newly developing immune checkpoint inhibitors (ICPi) that function by regulating T-cell activation^58^. Both PD-1 and PD-L1 are expressed on the surfaces of immune effector cells, such as T-lymphocytes, B-lymphocytes, NK cells, and other TILs. PD-1/PD-L1 interactions inhibit T-cell inflammation and other immune-mediated responses during infection. PD-L1 is also expressed in tumor cells and inhibits T-cell responses through up-regulating and binding PD-L1 to PD-1 to activated T-cells, thus resulting in immune consumption and diminishing the local immune response^59^. The expression of PD-L1 is associated with more advanced pathological grades, larger tumors, and loss of hormone receptors. Approximately 20%–50% of all TNBC hypotypes exhibit expression of PD-L1^60^. In July 2014, nivolumab, the first regulatory-approved PD-1 inhibitor, was applied in unresectable melanoma treatment. Other agents, such as the anti-PD-1 antibodies pembrolizumab and nivolumab, and anti-PD-L1 antibodies atezolizumab (MPDL3280A), durvalumab, and avelumab, have been shown to be effective in multiple TNBC clinical trials^61,62^. Next, we will discuss the better-researched drugs pembrolizumab and atezolizumab in TNBC immunotherapy.

The anti-PD-1 monoclonal antibody pembrolizumab received initial FDA approval for unresectable or metastatic melanomas in 2014^63^. Recently, a single-arm phase IB study in a cohort of 32 patients with PD-L1 recurrent or metastatic TNBC has evaluated the safety and clinical efficacy of pembrolizumab^64^. Pembrolizumab has been found to be well tolerated in intravenous injection at the dose of 10 mg/kg every 2 weeks; 47% of patients received more than 3 lines of treatment, and 21.9% received 5 or more treatments. In addition, Adams et al.^65^ have conducted a phase II study of pembrolizumab’s therapeutic effects in metastatic TNBC at a dose of 200 mg given once every 3 weeks for up to 2 years. Cohorts A and B were presented in the oral session at the 2017 ASCO meeting. Cohort A was defined as having confirmed metastatic TNBC with one or more systemic therapies for metastatic disease and progression on or after the most recent therapy. Of the 170 patients in the cohort, 61.8% had PD-L1-positive tumors, and 42.5% had received more than 3 previous lines of therapy for metastatic disease. The objective response rate (ORR) was 5.3% in the total cohort and 5.7% in the PD-L1-positive cases. The disease control rate (95% CI) was 7.6% and 9.5%, respectively. The researchers demonstrated that pembrolizumab has permanent anti-tumor effects and a controllable safety profile in metastatic TNBC. In the B cohort of this phase II study, Adams et al.^66^ evaluated pembrolizumab in a first-line therapeutic schedule in women with PD-L1-positive advanced TNBC as defined by an IHC-based composite score and no prior systemic therapy. Cohort B included 84 patients; 4 cases had a complete response, and 14 had a partial response, for a total ORR of 21.4%. The increased response in cohort B demonstrates the positive effects of applications of embrolizumab as a first-line therapy for PD-L1-positive advanced TNBC.

Recently, many studies have shown that pembrolizumab plus chemotherapy may be an effective neoadjuvant treatment for metastatic TNBC. In the phase III KEYNOTE-522 trial, patients receiving pembrolizumab in combination with chemotherapy before surgery were more likely to have a pathologically complete response than patients who received chemotherapy alone, regardless of PD-L1 levels^67^. Vinayak et al.^68^ have conducted an open-label clinical trial of niraparib combined with pembrolizumab for treatment of advanced or metastatic TNBC. The study enrolled 55 eligible patients, all of whom were administered 200 mg of oral ninraparib once daily in combination with 200 mg of intravenous pembrolizumabon once every 3 weeks. Of the 55 cases, 5 patients had confirmed complete responses, and 5 had confirmed partial responses (21% ORR). Fifteen of these cases were evaluable patients with tumor BRCA mutations, the ORR included 7 cases, and the median PFS was 8.3 months. A combination of niraparib with pembrolizumab shows promising anti-tumor activity in patients with advanced TNBC, with greater response rates in patients with BRCA mutations. A similar small cohort of patients in the KEYNOTE-173 suggested an improved ORR of 100% through treatment with pembrolizumab plus carboplatin *vs.* 80% in another experimental group with treatment with pembrolizumab plus nab-paclitaxel^69^.

Atezolizumab is a humanized immunoglobulin G1 isotype monoclonal antibody that targets PD-L1, which is expressed on the surfaces of tumor cells. Atezolizumab prevents PD-L1 binding to its receptors PD-1 or CD80, and then inhibits the PD-1/PD-L1 axis, thus preventing T-cell anti-tumor immunity. Atezolizumab as a new ICPi targeting PD-L1, thus providing effective and well-tolerated treatment options for metastatic TNBC. Patients receiving atezolizumab treatment show long-term clinical benefit. Patients with TNBC with high levels of TIL and PD-L1 expression appear to acquire enhanced clinical benefit. Emens et al.^70^ have performed a phase I study in a cohort of 116 patients with advanced TNBC to study and assess the safety and clinical efficacy of atezolizumab as a single agent therapy. They assessed the expression of PD-L1 in immune cells as well as in tumor cells by immunohistochemistry. The positive rate of PD-L1 expression in immune cells and tumor cells used the following scoring system: the percentage positivity of PD-L1 in immune cells was scored as “3” (> 10%), “2” (5%–10%), “1” (1%–5%), and “0” (< 1%). PD-L1 expression on tumor cells was classified into the following categories: “0” (less than 1%) and “1/2/3” (at least 1%). The ORR in all cases was 10%, and the median PFS and overall survival (OS) were 1.4 and 17.6 months, respectively. Compared with the patients with TNBC with 1% of PD-L1 expression on immune cells, the patients with PD-L1 expression of at least 1% on immune cells had significantly higher response rates and OS. Furthermore, higher expression of TILs was independently associated with a higher OS and longer ORRs. Adams et al.^71^ have conducted an additional phase IB trial in which 33 patients receiving chemotherapy for metastatic TNBC were studied to ascertain the therapeutic effect of atezolizumab in combination with nab-paclitaxel. In theory, the combination of ICPi with chemotherapy could expose higher levels of tumor-associated antigens to the immune system and then activate tumor-specific immunity to kill more tumor cells. The ORR in the 33 patients was 39.4%, whereas the median PFS and OS were 5.5 and 14.7, respectively. This research shows that a treatment program of atezolizumab combined with nab-paclitaxel is a valid therapeutic strategy for patients with TNBC. Many other clinical trials studying the therapeutic effects of atezolizumab in metastatic TNBC are ongoing, but the efficacy of aterzolizumab in TNBC requires further follow-up.

### PI3K/AKT/mTOR pathway

Aberrations in the PI3K/AKT/mTOR pathway are among the most common genomic abnormalities in various types of breast cancer^4,72,73^. Although PI3K/AKT/mTOR-targeted therapeutic schedules support early, direct targeting of this signaling pathway, clinical trials have not yet been successfully developed for TNBC.

PIK3CA, the most common activated isoform in TNBC, has various types such as α, β, δ, and γ subunits^74^. Buparlisib (BKM120), a PI3K inhibitor with activity resistance to all isoforms of PI3K, has already been evaluated by Baselga et al.^75^ in a phase III trial. Their results show that, in a total of 1147 post-antiestrogen therapy luminal breast cancers treated with buparlisib combined with fulvestrant, there was a higher PFS than that with cancers treated with fulvestrant alone. Pictilisib, another pan-PI3K antagonist, has a stronger inhibition effect toward the α-subunit than buparlisib^76^. Bupailisib shows a favorable toxicity profile; diarrhea and skin rash were the most common adverse reactions in randomized phase I/II trials^77,78^. The results of these studies illustrate that reagents with greater PI3K specificity may result in higher frequencies of adverse reactions than less potent reagents. In addition, more PI3K inhibitors include alpelisib (a specific α-subunit PI3K inhibitor) and serabelisib (a selective PIK3CA inhibitor)^79–81^. Various isoform-specific PI3K inhibitors are being tested in early phase clinical trials, but the clinical trial data on these drugs in TNBC are insufficient, and further study is needed. The important roles that PI3K isoforms play in TNBC progression suggest that PI3K antagonists might be an adjuvant therapy that could be combined with other chemotherapy strategies.

Approximately 70% of patients with TNBC harbor phosphorylated mTOR (p-mTOR); high expression of p-mTOR is significantly correlated with poor prognosis^82^. The mTOR inhibitor rapamycin shows approximately 77%–99% anti-tumor effects in a patient-derived xerograph TNBC model test, values significantly greater than those observed with doxorubicin^83^. Recently, several mTOR inhibitors have been applied in clinical trials. A phase I trial included 52 patients with advanced TNBC treated with doxorubicin, bevacizumab, and temsirolimus or everolimus^84^. The ORR was 21%, and the clinical benefit rate at 6 months was 40% among unselected patients.

On the basis of these original studies, new elements targeting various molecules of the PI3K/AKT/mTOR pathway continue to be developed. For example, gedatolisib can target and inhibit PI3K and mTOR and is more effective than everolimus, the currently approved mTOR inhibitor^85^. Gedatolisib significantly inhibits growth and proliferation of the TNBC cell line MDA-MB-231^76^. In an early phase clinical trial investigating endometrial cancer, gedatolisib was assessed for safety and antitumor activity, and the drug appeared to have a favorable toxicity profile^86^. Apitolisib is another equipotent PI3K/mTOR inhibitor. A preclinical trial has demonstrated that apitolisib inhibits more than half the tumor growth in nude mice injected with MDA-MB-231 TNBC cells^87^. Although many other dual PI3K/AKT/mTOR inhibitors continue to be discovered and studied in preclinical research, few clinical trials have tested these drugs in TNBC, and further studies are thus necessary.

### AMP-activated protein kinase (AMPK)

AMPK is a crucial metabolic energy sensor that regulates the protein and lipid metabolism response to changing energy supplies^88^. Many studies have shown that inhibition of the AMPK signaling pathways significantly influences the tumor growth and survival of patients with TNBC^89^. AMPK has a relatively high expression level in TNBC compared with non-TNBC tissues or cell lines. AMPK expression is correlated with TNM stage, distant metastasis, Ki-67 status, and shorter OS and DFS^90^. However, recent evidence indicates that pAMPK expression is approximately 90% lower in tumor tissues than normal breast epithelial cells, and this decreased pAMPK is correlated with higher histological grades and axillary node metastasis^89^.

Much evidence supporting metformin as a first-line treatment for type 2 diabetes mellitus also suggests that it can reduce breast cancer risk^91^. Metformin activates the AMPK pathway by inhibiting complex 1 of the mitochondrial respiratory chain, thus resulting in the suppression of mTOR and hence a decrease in cell proliferation and the repression of glucose synthesis^92^. As detailed in terms of apoptosis, TNBC cell lines are more sensitive to metformin than non-TNBC cell lines^91^. Although metformin has anti-TNBC effects *in vitro*, its efficacy remains to be substantiated in clinical studies. The compound 5-aminoimidazole-4-carboxamide ribose (AICAR) is another pharmacologic AMPK activator, though its mechanism is different from that of metformin^93^. AICAR, through activation of AMPK phosphorylation, promotes arrest of TNBC cell growth and inhibits TNBC tumor migration or invasion^94^. Although data concerning AICAR in clinical trials of patients with TNBC are lacking, these findings demonstrate the important role of AMPK in regulating oncogenes.

Demethoxycurcumin (DMC) is a structural analogue of curcumin that has antioxidative, anti-inflammatory, anti-tumor, and anti-angiogenesis effects^95^. DMC has no pharmacokinetic effect on normal mammary tissues, and the most effective cytotoxic effects of DMC are on TNBC cells, as compared with other breast cancer types^96^. Shackelford et al.^97^ have demonstrated that DMC suppresses TNBC cell proliferation and inhibits many carcinogenic signaling pathways *via* active AMPK phosphorylation at low micromolar levels. In addition, DMC-mediated AMPK phosphorylation promotes the degradation of EGFR, which exhibits high expression in TNBC and plays a role in promoting tumors^98^. Furthermore, DMC targets various AMPK downstream targets and hinders activation of the oncogene STAT3^99^. In addition, fluoxetine, through activation of AMPK-mTOR-ULK signaling, has been suggested to exert anti-tumor effects, thus leading to the conclusion that AMPK may be a novel anti-TNBC target^100^. In fact, AMPK function is impaired in primary TNBC, and loss of AMPK signaling in patients usually indicates poor prognosis, thereby suggesting that AMPK reactivation has potential in TNBC prevention and treatment. Some researchers believe that AMPK phosphorylation may have an anticancer role in TNBC progression^101^. AMPK phosphorylation has anti-TNBC effects because of its targeting and inhibition of AKT/mTOR signaling; various AMPK activators, such as metformin, AICAR, DMC, and fluoxetine, also confirm this conclusion. AMPK should be considered as a crucial therapeutic target for TNBC, and further pre-clinical and clinical research on AMPK agonists in anti-TNBC effects is essential.

## Conclusions

Although typical TNBC commonly has poor prognosis, several rare pathological types of TNBC are different from typical TNBC and have relatively satisfactory prognosis. However, carcinoma with apocrine differentiation does have relatively poor prognosis, as compared with that of NST invasive carcinomas, possibly because of the high expression of AR in this type of breast cancer; usually, this subtype of breast cancer without HER2 expression has high overlap with the LAR subtype. The androgen signaling associated with these carcinomas may lead to new treatments for these tumors in the future. Many studies have demonstrated that positive AR expression increases cancer cells’ chemotherapy resistance and stem cell-like properties. AR-targeted treatment has shown promising preliminary results in TNBC, and AR inhibitors such as bicalutamide and enzalutamide have been sufficiently assessed in preclinical and/or clinical studies. Most AR-targeted genes or interacting pathways have been studied in prostate cancer, whereas understanding of AR signaling pathways in breast cancer and especially in TNBC remains relatively limited. More extensive study of the heterogeneity of AR in breast cancer is necessary.

**Figure 2:**
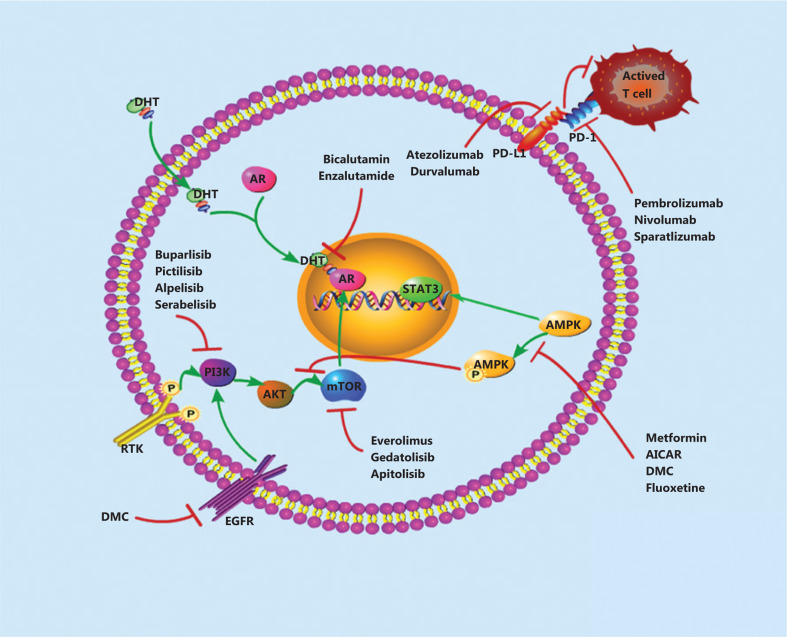
A model showing current therapeutic strategies that could be combined with other therapies. The therapeutic strategies include AR-inhibitors, PI3K/AKT/mTOR inhibitors, AMPK inhibitors, and immunotherapy.

In early-phase trials, studies have demonstrated that in unselected PD-L1-positive tumors, the response rates with anti-PD1 or anti-PD-L1 treatment can increase up to 10%, and slight improvements can result in rates of 20%–30%. Beyond the immunosuppression regents introduced in this paper, additional new immune checkpoints are being analyzed in early phase trials, and tumor vaccine strategies for TNBC are increasingly causing excitement in the field. In addition, the development of drugs targeting the PI3K/AKT/mTOR or AMPK pathways for TNBC treatment described herein are a gradually developing field in which the efficacy and toxicity of new agents, in addition to their interactions with different cancer pathways, should be considered. A summary of the above new targets for TNBC is shown in **Figure 2**. With the development of molecular research, driver mutations in this disease will be better understood, and more targeted treatments could become available for patients with TNBC.

## Figures and Tables

**Table 1:** Summary of rare triple negative breast cancers

Variables/population	Adenoid cystic carcinoma	Secretory carcinoma	Acinic cell carcinoma	Carcinoma with apocrine differentiation	Carcinoma arising in microglandular adenosis
ER	Negative	Negative	Negative	Negative	Negative
PR	Negative	Negative	Negative	Negative	Negative
HER2	Negative	Negative	Negative	Positive/Negative	Negative
AR	–	–	Negative	Positive	–
Mean age (range)	64 years	25 years (3–87 years)	56 years (35–80 years)	–	50–60 years
Location (percentage)	Subareolar (50%)	Near the areola	–	–	–
Tumor size (average)	0.5–12 cm (3 cm)	0.5–12 cm (3 cm)	1–5 cm	–	Typically a microscopic lesion
Chromosomal translocation	t (6; 9) (q22-23; p23-24)	t (12; 15)	–	Gain of 1p, 1q, 2q, loss of 1p, 12q, 16q, 17q	Gain of 8q, loss of 5q
Fusion/mutation genes	MYB, NFIB	ETV6-NTRK3	TP53, MLL3, FOXA1	AR	–
Malignant biological properties	Low-grade malignant	Low-grade malignant	Low-grade malignant	–	Uncertain
Prognosis	10-year survival rates > 90%	Favorable prognosis in young patients (< 20 years)	Favorable prognosis	Relatively poor prognosis	Uncertain
